# Is 70 the new 60? A longitudinal analysis of cohort trends inintrinsic capacity in England and China

**DOI:** 10.21203/rs.3.rs-4271576/v1

**Published:** 2024-05-30

**Authors:** John Beard, Hanewald Katja, Yafei Si, Jotheeswaran Thiyagarajan, Dario Moreno-Agostino

**Affiliations:** Columbia University - Mailman School of Public Health; University of New South Wales; The University of New South Wales; World Health Organization; University College London

## Abstract

To understand how the health of older adults today compares to that of previous generations, we estimated intrinsic capacity and subdomains of cognitive, locomotor, sensory, psychological and vitality capacities in participants of the English Longitudinal Study on Ageing (ELSA) and the China Health and Retirement Longitudinal Study (CHARLS). We applied multilevel growth curve models to examine change over time and cohort trends. We found that more recent cohorts entered older ages with higher levels of capacity, and their subsequent age-related declines were somewhat compressed compared to earlier cohorts. These improvements in capacity were large, with the greatest gains being in the most recent cohorts. For example, a 68-year-old ELSA participant born in 1950 had higher capacity than a 62-year-old born just 10 years earlier. Trends were similar for men and women, and findings were generally consistent across English and Chinese cohorts.

## INTRODUCTION

Over the past century, life expectancy has risen in almost every country, and longer lives are increasingly becoming the norm.^1^ Initially, this trend was driven by increased survival through childhood and childbirth, but in more developed countries, it is now mainly a consequence of longer survival at older ages.^2^ Yet, while these improvements in life expectancy are well documented, it is still uncertain how the health of older people today compares to that experienced by previous generations at similar ages.^3–6^

One reason for this uncertainty is that health is a multifaceted concept, and no consensus has been reached on how to frame or measure it.^7^ Temporal trends are particularly difficult to interpret when considered from the perspective of disease.^6^ Broader access to effective health care means that some people who would previously have died from a condition now survive into older ages, and these improvements in survival have led to greater disease prevalence.^6^ However, the advances in medical care that increase survival may have also reduced the impact that these conditions have on people’s lives. Treatments for other conditions, too, may have lessened their influence. For example, a person with osteoarthritis of the hip, who might have previously experienced severe disability, may now regain high levels of locomotor capacity following joint replacement.^8^ Other people may live much of their lives free of disease but still experience declines in physical or mental capacities.^9^

Furthermore, older people often report that the health outcome they value most is not the presence or absence of disease, or even life extension, but their level of functioning and independence.^10^ Yet trends in functioning have also been difficult to assess. For example, to explore changing patterns of disability, the Global Burden of Disease group has applied disability weightings to trends in disease prevalence. This analysis suggests that health-adjusted life expectancies are not keeping pace with increases in life expectancy.^11^. However, these disability weights are only indirect, generic estimates of a disease’s impact on functioning and cannot account for geographic or temporal variations in access to health care or other resources that may mitigate the consequences of these conditions.^12^

Meanwhile, most of the research that directly estimates functional trends has been limited to measures of severe disability, such as Activities of Daily Living (ADL) or Instrumental Activities of Daily Living (IADL). The findings of these studies have been varied and inconclusive.^13–17^ Moreover, since loss of ADLs or IADLs only becomes apparent after very significant declines in functioning, these categorical outcomes tell us little about the functional status of the far broader population of older adults who have not experienced these major losses.

The World Health Organization (WHO) has proposed a framework for understanding ageing and health that allows a more nuanced consideration of trends in functioning and health across the full range of ageing populations. In this model, healthy ageing is considered not from the perspective of disease but based on an individual’s ability to be and do the things they value.^1^ This ability is understood to be determined by individual-level attributes – a person’s “intrinsic capacity”, as well as the environments they inhabit and the interaction between the individual and these environments.^18^ Since intrinsic capacity is framed as a continuum that can be considered across the second half of life, it can potentially be used to compare incremental changes among both relatively robust and severely disabled individuals.

We have previously examined intrinsic capacity in two large longitudinal studies of the English and Chinese populations: the English Longitudinal Study on Ageing (ELSA)^9^ and the China Health and Retirement Longitudinal Study (CHARLS).^19^ Both analyses identified an intrinsic capacity construct comprising subdomains of cognitive, locomotor, sensory and psychological capacity and a further subdomain labelled vitality, which may represent underlying age-related biological changes and energy balance. This structure is consistent with what had been previously suggested by gerontological theory.^20^ Our analyses showed intrinsic capacity to have strong construct validity and to be a powerful predictor of subsequent care dependence even after adjustment for multimorbidity, age, gender and socioeconomic status. Subsequent research has shown it to also predict mortality and specific conditions.^21^

The aim of this paper is to conduct a longitudinal analysis of cohort trends in intrinsic capacity in these same studies to determine whether older adults in England and China are experiencing the same, better or worse capacity than people of similar ages in the past. We undertook secondary analysis of data from ELSA using multilevel growth curve models to identify period and cohort trends in intrinsic capacity and its subdomains. We then applied the same methods for a comparative analysis of the CHARLS cohort.

## RESULTS

### Main analyses – ELSA

The sample for the main analyses (ELSA) included 14,710 different participants aged 60+, including 53.3% women (n = 7,841). The median birth year was 1940 (interquartile range, IQR: 1931–1948), and the median number of observations was 4 (IQR: 2–6).

CFA models with a bifactor structure consisting of intrinsic capacity and five subdomains (cognitive, locomotor, sensory, psychological and vitality, as in Beard et al. 2019), led to non-significant loadings of the locomotor indicators on their subdomain, suggesting the collapse of this specific factor once the common factor (intrinsic capacity) had accounted for the common variance across these indicators. Therefore, the bifactor model was respecified, removing the “locomotor” subdomain. The updated configural bifactor model in ELSA showed good fit (Table 1, **upper section**). Additional equality constraints to the loadings and thresholds across time points did not lead to a loss in fit (rather, fit increased due to the increased model parsimony). Based on these results, scalar invariance of the proposed intrinsic capacity bifactor model was assumed to hold, and similar evidence was found for the correlated factors model, thus enabling comparisons of the levels in the intrinsic capacity factors and all the different subdomains over time.

**Figure 1:**
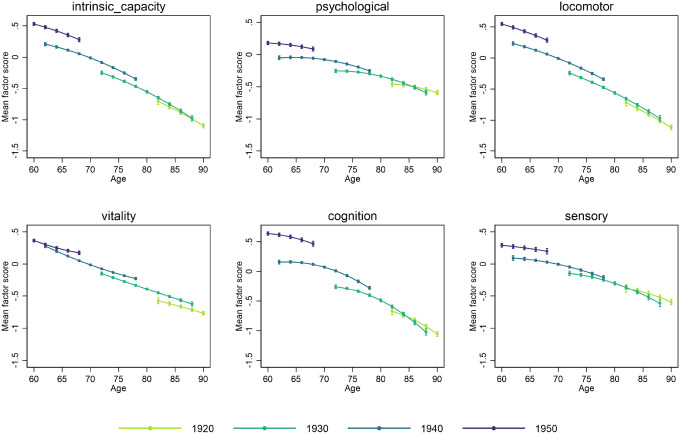
Intrinsic capacity and subdomains mean factor scores by birth cohort and age in ELSA (main analyses). Note: Longitudinal individual weights were used for multilevel growth curve models to ensure representativeness to participants aged 50+ and living in England in 2002 who were still alive and residing in private households by wave 9. The point estimates and their 95% confidence intervals are displayed in the graph.

Factor scores were then derived from the bifactor model (intrinsic capacity) and correlated factor models (psychological, locomotor, vitality, cognitive, and sensory), including all observations with at least partial information between waves 1 and 9 of ELSA. The results of the multilevel growth curve models computed using these scores are shown in Table 2 **(left section)**, and the corresponding marginal mean predicted levels for different birth cohorts are plotted by age in Fig. 1. All confidence intervals (CI) were constructed at a 95% confidence level.

More recent cohorts entered older ages with significantly higher levels of intrinsic capacity (Birth year: 0.046 (CI: 0.043–0.048), p < 0.001). While intrinsic capacity levels declined with increasing age across all cohorts, these declines were less steep for more recent cohorts than for earlier cohorts (linear change * birth year: 0.001 (CI: 0.000–0.002), p = 0.004).

More recent cohorts also entered older ages with significantly higher levels for each subdomain of capacity, with these improvements being largest in the locomotor, vitality and cognition subdomains. As with intrinsic capacity as a whole, declines with increasing age were observed across all subdomains. However, declines in locomotor and cognitive subdomains were less steep among more recent cohorts. For vitality, they were initially faster among younger cohorts (linear change * birth year: −0.001 (CI: −0.002–0.000), p = 0.001) but subsequently followed more stable levels over time (Quadratic change * birth year: 0.000 (CI: 0.000–0.000), p = 0.002).

To quantify the observed improvements shown in Fig. 1, we calculated the marginal mean predicted levels for each age by cohort (Table 3 **and Supplement 4**). Even when comparisons are limited to cohort participants for whom data is available at the same age, the observed improvements are large. For example, intrinsic capacity of the cohort born in 1950 at age 68 was 0.280 (CI: 0.248–0.313), significantly higher than the 0.208 (CI 0.183–0.233) of the cohort born in 1940 at age 62. The pattern was similar for psychological, locomotor, cognitive and sensory capacities, although the improvement was most pronounced for cognitive capacity. The greatest improvements were between the most recent (1950) cohort and the 1940 cohort, although direct comparisons between earlier cohorts also showed significant improvements in the more recent cohort **(Supplement 4)**. If these directly observed trends were extrapolated to compare the earliest with the most recent cohort, the improvements would be significantly greater than those we could observe directly.

### Comparative analyses – CHARLS

The sample for the comparative analyses (CHARLS) included 11,411 participants aged 60+, including 50.0% women (n = 5,706). The median birth year was 1947 (IQR: 1941–1951), and the median number of observations was 2 (IQR: 1–3).

The results from the measurement invariance testing (Table 1, **lower section**) suggested that scalar invariance held for the bifactor model (intrinsic capacity). However, the restrictions imposed to fit the scalar model in the correlated factors model resulted in a substantial loss in fit according to the change in CFI (change in RMSEA was within boundaries). Factor scores for each subdomain were derived from but interpreted with additional caution in the case of the subdomains, as changes in the score levels could be due to differences in the measurement parameters across the time points. Therefore, trajectories in the subdomains for the CHARLS need to be considered with caution.

**Figure 2:**
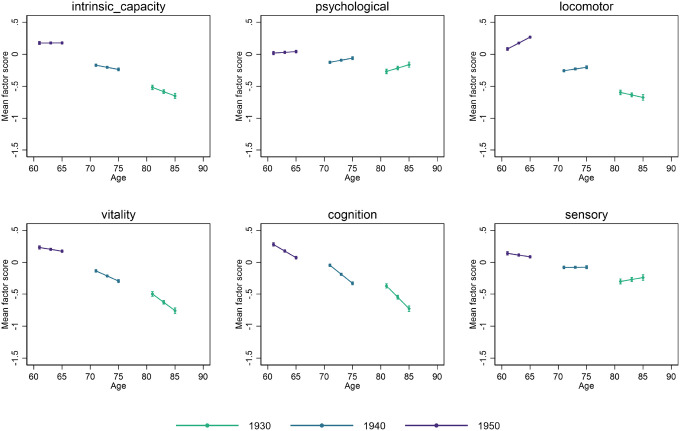
Intrinsic capacity and subdomains mean factor scores by birth cohort and age in CHARLS (comparative analyses). Note: Survey weights were used to ensure representativeness to participants aged 50+ and living in China. Here we displayed the point estimates and their 95% confidence intervals from the multilevel growth curve models.

Coefficients from the multilevel growth curve models estimated with CHARLS data as part of the comparative analyses are shown in Table 2 (right section), and the corresponding marginal mean predicted levels for different birth years are plotted by age in Fig. 2. Consistent with the main analyses for ELSA, more recent cohorts entered older ages with higher levels of capacity (Birth year: 0.035 (CI: 0.032–0.037), p < 0.001). The largest improvements were found for the vitality subdomain, followed by the locomotor, cognition, sensory and, finally, psychological factors. Intrinsic capacity was found to decline significantly with age, and subsequent declines for more recent cohorts were less steep than for earlier cohorts (Linear change * birth year: 0.002 (CI: 0.001–0.002), p < 0.001). Findings for the subdomains may be at least partly attributed to the lack of measurement invariance of the subdomain analysis outlined above. As with the main analysis, steeper declines with increasing age were observed among earlier cohorts in locomotor, vitality, and cognition. However, for the psychological and sensory subdomains, changes over time were found to be positive with increasing age overall (Linear change), with more recent cohorts experiencing smaller improvements (Linear change * birth year).

### Sensitivity analyses

We performed sensitivity analyses in ELSA, replacing the longitudinal weights with cross-sectional survey weights and found similar results (Supplement 5). We also undertook additional analysis to determine whether the observed trajectories varied by gender. Measurement invariance did not hold across both waves and genders in either ELSA or CHARLS. However, measurement invariance was found to hold over time *within* genders in most cases except for the correlated factors model in CHARLS. Factor scores were therefore derived separately for each gender, and multilevel growth curve models were estimated by gender using the corresponding factor scores. Although direct comparisons across gender could not be made since they would be biased by differences in the measurement of the intrinsic capacity factor and its subdomains, within-gender trajectories mirrored those found in the overall analyses (Supplement 6).

## Discussion

Our research suggests there have been significant improvements in functioning in more recent cohorts of older people in both England and China. Within ELSA, more recent cohorts entered older ages with higher levels of intrinsic capacity, and subsequent declines were less steep than for earlier cohorts. Improvements were seen in all subdomains. Trajectories were similar for males and females and largely consistent across both countries, although our analysis was limited by the lesser availability of data waves in CHARLS.

The observed improvements are substantial. To avoid undue extrapolation, we limited our assessment to direct comparisons of capacity in participants of different cohorts at the same age. Currently, the overlap between adjacent cohorts in the ELSA study is 6 years, and participants of non-adjacent cohorts cannot be directly compared. However, even with these limitations, we still found that a 68-year-old ELSA participant born in 1950 had higher intrinsic capacity than a 62-year-old born just 10 years earlier. Improvement in cognition was even more substantial. When comparing earlier cohorts, additional improvements are observed, although the gains between these cohorts are not quite as large as between the 1940 and 1950 cohorts. Thus, while our models suggest that today’s 70-year-olds have the equivalent functioning to substantially younger adults in previous generations (perhaps 70 really is the new 60), our direct assessments can only confirm that 68 is the new 62.

These observed improvements stand in contrast to previous research, which has found that increases in longevity have been accompanied by increased prevalence of chronic conditions in older age groups.^6^ This increased prevalence is likely driven, at least in part, by people who would have previously died from a condition such as heart disease now surviving into older ages. However, since the management and functional consequences of chronic conditions may also have changed, the implications of these trends on the day-to-day lives of older adults have been less clear.

Most previous research directly examining functional trends has been limited to studies of severe disability, and the findings have been inconsistent. For example, in the UK, a comparison of similar cohorts of people over age 65 between 1991 and 2011 suggested that only 36.4% of the extra years of life gained for men and 4.8% for women were experienced with no level of care dependency.^13^ On the other hand, analysis of ELSA data from 2002 to 2016 found that ADL limitations declined in those aged 55 to 64 years.^14^ In China, some studies have suggested the age-adjusted prevalence of ADL loss may be declining^16^, while others found that limitations in ADLs and IADLs may be increasing^17^ or that there may be a V-shaped trend for ADLs.^15^

These inconsistencies are likely to arise partly from the wide variety of measures used.^22^ Some measures (particularly IADLs) also have difficulty distinguishing between changes that may be occurring in the individual and those that might result from changes in the environment. For example, a common IADL question relates to how easy it is to use a phone, yet phone type and use have changed with time.^23^.

Another important influence on these past findings could be changing patterns of institutionalisation. In the UK, the number of nursing home beds per 100 people aged 75 and over fell by 12% between 2012 and 2022, and admissions for those aged 65 and over fell by 18% between 2014 and 2022.^24^. As older adults became less likely to be cared for in institutions and more likely to be cared for at home, the prevalence of severe disability in community-based samples would increase, even if the prevalence remained unchanged in the total population. Changing patterns of institutionalisation may have influenced our findings as both studies use community-based samples. However, the shift in institutionalisation in England would operate against the positive trends we observed, while in China, institutionalisation rates remain low at around 1%, and recent emphasis has been on community-based care services.^25^

Furthermore, the most fundamental limitation of studies of severe disability is that they cannot assess how increasing life expectancy and associated changes in disease prevalence might be impacting on the functioning of people in relatively robust health. This is where our findings shed new light. In contrast to previous research, the continuous nature of the intrinsic capacity construct and its subdomains allowed us to examine milder and earlier forms of age-related disability than previous analyses, and to consider individual-level changes independent of any changes that may have occurred in contextual factors.

The improvements in functioning that we identify could arise from multiple influences and have no obvious single driver. Greater access to healthcare or improved treatments may have played a role. Detection and management of biological risk factors may also have improved, reducing their impact (and potentially increasing their prevalence), but observed trends are inconsistent. In ELSA, rates of awareness of hypertension, treatment of hypertension, and the proportion of treated participants who achieved recommended targets have increased over time.^26^ Management of hypertension in China has also improved, although the age-standardised prevalence of high blood pressure appears to have increased significantly.^27^ A reported increase in the prevalence of diagnosed diabetes in ELSA participants from 7.7% in 2004 to 11.5% in 2012 would also be consistent with better detection and possibly management.^28^ However, between 2004 and 2012, there was also a significant rise in the prevalence of undiagnosed diabetes and only a very small decrease in the proportion of participants with diabetes who were unaware of this condition.^28^

Another possibility is that healthier behaviours may have slowed age-associated biological changes, strengthening biological reserves and limiting the impact of chronic conditions. However, if this were a major influence, it would likely also have served to reduce the incidence of chronic disease, which runs counter to observed trends. Moreover, behaviours and risk factors in the UK and China have trended in multiple directions over the past 25 years. Age-standardised prevalence estimates suggest that between 1990 (or 2000, depending on data availability) and 2015, tobacco use fell in the UK but remained relatively steady in China, while the prevalence of being overweight rose in both countries.^29^ Trends in physical activity in the UK and elsewhere are hard to determine, but may have declined over time.^30,31^ In China, physical activity from work and domestic activities may have fallen by around 50% between 1991 and 2011,^32^ although other analyses suggest a more stable long-term trend in China, at least from 2000 to 2015.^33^

Other explanations for the observed trends may lie in the more distant past. The cohorts included in these studies were born between 1920 and 1959 for ELSA and 1930 to 1955 for CHARLS, and early life experiences from these periods, which include World War II and the Chinese Civil War, may have played a role.^34^ Our own research in CHARLS has previously shown that early life events such as poor nutrition account for 16% of the variance in capacity observed in older adults in China.^35^ Even the antenatal experience of mothers may influence the risk of chronic conditions in their children.^36,37^

Higher early life peaks in capacity are likely to also provide greater reserves for people to draw on as they age, delaying overt declines in capacity. For example, greater educational opportunities in childhood have been suggested as one explanation for the 13% per decade decline in the incidence rate of dementia observed in Europe and North America over the past 25 years.^38,39^ In our analysis, more recent cohorts entered older age with higher capacity, and this would be consistent with an influence of early life factors such as education or nutrition.

Finally, one hypothesised cause of multiple chronic conditions is inflammaging, which is thought to be driven by multiple factors, including infections and nutrition.^40^ Changes in exposure to common pathogens across the life course related to better sanitation and other environmental factors could thus also have played a role.

In summary, the explanations for the improvements we have observed are likely to be complex and relate to changes that have occurred over most of the past century.

Our analysis has many strengths, including the representative nature of the samples. The instruments underpinning our measure are widely used and, where possible, objective. They distinguish between individual-level change and changes that might have occurred in the physical and social environments the individual habits.

However, when considering these findings, it is important to understand the limitations of our research. We explored the typical experience of cohorts, and this is likely to mask significant intra-cohort heterogeneity. We considered this possibility in our gender analysis which suggested the improvements we observed were not limited to one sex. However, previous research suggests that positive trends are likely to be greater for more advantaged socioeconomic groups, and we cannot exclude this possibility.^41,42^

It is also likely that participants with worse intrinsic capacity were disproportionately excluded from the study samples, particularly for older ages and cohorts. However, any resulting survivor bias would likely be greater for older cohorts, and any effect would be to underestimate the positive trends we observed.

Due to the complexity of the measurement models, we could not embed the latent variables themselves in the analyses of the longitudinal trajectories. Rather, we derived factor scores and analysed these over time. These factor scores are assumed to be free of error (as would be any other observed outcome), so it is important to acknowledge that measurement error may still be a source of bias in this study.

It is also possible that self-report effects may be at play in the psychological and sensory subdomains, and trends in the sensory domain may have been impacted by changes in access to hearing and visual supports. However, the steepest improvements in capacity were found in subdomains measured with objective indicators, suggesting they are not explained by reporting bias. Finally, attrition within the two studies also needs to be considered as a possible influence on our findings. However, sample attrition in ELSA has been previously shown not to significantly affect estimates of disease prevalence, suggesting any influence is likely to be minor.^43^

Our findings suggest several avenues for further research. If they can be replicated and the limitations addressed, future studies could examine whether trends vary between settings, how trends might be influenced by socioeconomic and other characteristics, such as race or ethnicity, and why these trends may be occurring. This might suggest interventions to ensure the trends we have observed are reinforced and equitably spread.

In the meantime, our analysis strongly suggests that increasing life expectancy is being accompanied by large increases in health expectancy among more recent cohorts, at least when focusing on people born between 1920 and 1959 This has positive implications for all of us, both as individuals and for society more broadly.

## Online Methods

### Sample

ELSA follows a nationally representative sample of the English population aged 50 and above, while CHARLS follows a nationally representative sample of the Chinese population aged 45 and above. Data collection in both cohorts was conducted through face-to-face assessments using computer-assisted personal interviews. In addition, objective measures and blood samples were collected by trained nurses in waves 2, 4 and 6 in ELSA and waves 1 and 3 in CHARLS. The response rates in both ELSA and CHARLS were reasonably high, although they varied across waves. The average follow-up length is about 4.84 years in ELSA and 2.18 years in CHARLS, with attrition rates of 36.3% in ELSA and 45.0% in CHARLS from wave 1 to wave 2. The details regarding follow-up information and missing information are provided in Supplement 2.

We included ELSA and CHARLS participants aged 60 + with valid information in at least one of the indicators used to measure intrinsic capacity in at least one wave. ELSA currently has nine waves of data available, while five waves are available for CHARLS. Because of the comprehensive measures included in this longitudinal study and the many years of follow-up, we made ELSA [waves 1 (2002) to 9 (2019)] the focus of our main analysis. We then applied the same methods to the CHARLS [waves 1 (2011) to 3 (2015)] cohort but given the shorter follow-up period, we report this as a comparative analysis. More recent data from CHARLS [waves 4 (2018) and 5 (2020)] were not included as, by design, none of the locomotor and vitality subdomain indicators was assessed. This research involved secondary analysis of previously collected data, and patients and the public were not involved in any way.

### Measures

We used data from multiple self-reported and objectively measured tests to create scores for intrinsic capacity and subdomains of capacity consistent with the WHO model of intrinsic capacity.^20^ To maximise the comparability across the two cohort studies, we focused on the indicators that were present in both ELSA and CHARLS (see Supplement 1 for details and descriptive results).

Building on our previous analyses, we used a confirmatory factor analysis (CFA) approach to operationalise a set of relevant subdomains.^9,19^ These comprised locomotor capacity (measured by walking speed, chair-stand test and balance), cognitive capacity (immediate recall, delayed recall, time orientation/memory), sensory capacity (reported hearing and visual impairments), psychological capacity (affect and sleep as measured by Center for Epidemiological Studies-Depression (CES-D) scale^44^ items present in ELSA and CHARLS) and vitality (grip strength, forced expiratory volume, haemoglobin). Intrinsic capacity was operationalised as a latent common cause of the levels across all indicators (general factor under a bifactor structure) after accounting for the unique shared variance among subsets of indicators as captured by the subdomains. A visual depiction of the measurement model is available in Supplement 3.

### Statistical analyses

To ensure that the constructs (i.e., the intrinsic capacity general factor and the subdomains) under study were equivalently measured over time, we first used a measurement invariance testing approach.^45,46^ In this approach, a multiple groups CFA model without constraints (i.e., configural model) was rst estimated to assess whether the same factor structure held across time points (i.e., configural invariance). Configural invariance was deemed to hold if the values of the root mean square error of approximation (RMSEA) and the Tucker-Lewis Index (TLI) for the configural model were below 0.060 and above 0.950, respectively^47^. Provided that configural invariance held, an additional level of invariance, scalar invariance, was tested, where factor loadings and item intercepts/thresholds were fixed to be equal across time points. Scalar invariance ensures that comparisons of the levels in the constructs are not biased due to differences in the way in which they are measured across time points^45^. Since the main aim of this study was to explore the trajectories in those constructs, ensuring that scalar invariance held was crucial. Scalar invariance was deemed to hold if the difference in t between the scalar and configural model was smaller than 0.015 and 0.010 in the RMSEA and the Comparative Fit Index (CFI), respectively^48,49^. These models were computed using the data from participants present in all the waves in which all indicators were present (i.e., waves 2, 4, and 6).

Once measurement invariance had been tested in the waves in waves 2, 4, and 6 in ELSA and waves 1 and 3 in CHARLS, the measurement models were extended to include the remaining waves (waves 1, 3, 5, 7, 8, 9 in ELSA, waves 2 in CHARLS), where only partial information was available by design. We used weighted least squares mean and variance adjusted (WLSMV) estimation with pairwise deletion to estimate these models and generate factor scores representing the individuals’ levels of intrinsic capacity and each of the subdomains based on the factor models with multiple indicators. The use of pairwise deletion allowed us to obtain estimates of the measurement models in the presence of partial information based on the pattern of relationships between the indicators across the waves, maximising the use of the information available for each individual.^50^ While this approach can provide biased estimates if the data are not missing completely at random, it retains more information from the available data, which maximises the reliability and validity of the model estimates and offers more plausible results compared to other approaches like listwise deletion.^50^ Using alternative estimation procedures with more plausible assumptions (e.g., full information maximum likelihood assuming data are missing at random) was not feasible due to the complexity of the measurement models.

Intrinsic capacity factor scores were derived from the bifactor models, while subdomain factor scores were estimated using correlated factors models where only the subdomains were present and allowed to correlate with each other. We used this approach for the subdomain factor scores because bifactor models would give ‘residualised’ versions of the subdomains, capturing what was left after accounting for the general factor.

Multilevel growth curve models were then used to model the change over time in intrinsic capacity and the five subdomains (psychological, locomotor, vitality, cognition, and sensory).^51^ Time was included in the models as the years elapsed since the first wave. Both linear (constant change) and quadratic (accelerated change) terms were included to allow for non-linear trajectories in ELSA. However, for CHARLS, only linear trajectories were analysed due to fewer repeated measurements available. Birth year (in years, centred at 1920 in ELSA and 1930 in CHARLS) was included in the models as a covariate to account for potential differences in the initial levels across cohorts. Interaction terms between birth year and the growth parameters (i.e., linear and quadratic) were included in the models to account for potential differences in the rates of change across cohorts. We acknowledged the heterogeneity in the intercepts and rates of change by modelling the random effects of both the intercepts and linear slopes, which were allowed to correlate. Variation in the rates of change over time was captured by the random effects for the linear/constant change (random effects for the quadratic/accelerated change could not be included due to model estimation/convergence issues).

All models were computed using survey weights to restore representativeness to each study’s population of reference. In the main ELSA analysis, to confirm the robustness of the results to the differential non-response to the different waves and to ensure representativeness to participants aged 50 + and living in England in 2002 and still alive and residing in private households by wave 9, we estimated the models using longitudinal weights.^52^ Because these longitudinal weights take into account the differential non-response to all the waves, analyses do not rely on the assumption of the data being missing completely at random but rather on them being missing at random after conditioning on the variables used to derive those weights. In CHARLS, survey cross-sectional weights were used for multilevel growth curve models to ensure representativeness to participants aged 50 + and living in China.^53^ []. All the weights are provided by the ELSA and CHARLS teams. To aid the interpretation of the results, marginal predicted levels were obtained from the models for each of the data collection time points and plotted in year and age vector plots. Furthermore, these marginal predicted levels were tabulated in age*cohort grids.

## Figures and Tables

**Table 1:** Results from the measurement invariance testing.

	Model	Invariance level	Chi-2 (df)	RMSEA (90% CI)	CFI	TLI	ΔRMSEA	ΔCFI
Main analysis (ELSA: n = 3,246)	Bifactor model	Configural	1691 (357)	0.034 (0.032, 0.036)	0.981	0.975		
		Scalar	1962 (441)	0.033 (0.031, 0.034)	0.978	0.977	0.001	−0.003
	Correlated factors model	Configural	1874 (375)	0.035 (0.034, 0.037)	0.978	0.973		
		Scalar	2374 (429)	0.037 (0.036, 0.039)	0.972	0.970	−0.002	−0.006
Comparative analysis (CHARLS: n = 5,57l)	Bifactor model	Configural	2378 (234)	0.041 (0.039, 0.042)	0.958	0.945		
		Scalar	2789 (289)	0.039 (0.038, 0.041)	0.951	0.948	0.002	−0.007
	Correlated factors model	Configural	2118 (253)	0.036 (0.035, 0.038)	0.963	0.956		
		Scalar	2967 (289)	0.041 (0.039, 0.042)	0.947	0.944	−0.005	−0.016

*Note*. df: degrees of freedom; RMSEA: Root Mean Square Error of Approximation; CFI: Comparative Fit Index; TLI: Tucker-Lewis Index; ΔRMSEA: difference in RMSEA; ΔCFI: difference in TLI. Scalar invariance is usually deemed to hold if the difference between the configural model in the RMSEA (ΔRMSEA) and the Comparative Fit Index (CFI) is smaller than 0.015 and 0.010, respectively. Configural invariance is usually deemed to hold if the values of the root mean square error of approximation (RMSEA) and the Tucker-Lewis Index (TLI) for the configural model were below 0.060 and above 0.950, respectively (24).

**Table 2:** Results from the multilevel growth curve models performed in ELSA and CHARLS

		Main analyses (ELSA)				Comparative analyses (CHARLS)			
		Coef.	[95% Conf.	Interval]	*p*-value	Coef.	[95% Conf.	Interval]	*p*-value
Intrinsic Capacity	Linear change	−0.041	−0.055	−0.027	< 0.001	−0.033	−0.044	−0.022	< 0.001
	Quadratic change	−0.001	−0.002	0.000	0.031				
	Birth year	0.046	0.043	0.048	< 0.001	0.035	0.032	0.037	< 0.001
	Linear change * birth year	0.001	0.000	0.002	0.004	0.002	0.001	0.002	< 0.001
	Quadratic change * birth year	0.000	0.000	0.000	0.945				
	Intercept	−0.706	−0.753	−0.659	< 0.001	−0.518	−0.551	−0.484	< 0.001
	*Slope variance*	*0.001*	*0.001*	*0.001*		*0.037*	*0.035*	*0.039*	
	*Intercept variance*	*0.397*	*0.376*	*0.419*		*0.572*	*0.551*	*0.593*	
	*Slope-intercept covariance*	−*0.010*	−*0.011*	−*0.008*		−*0.073*	−*0.079*	−*0.068*	
Psychological	Linear change	−0.004	−0.017	0.008	0.491	0.026	0.014	0.039	< 0.001
	Quadratic change	−0.002	−0.002	−0.001	< 0.001				
	Birth year	0.020	0.018	0.023	< 0.001	0.014	0.012	0.017	< 0.001
	Linear change * birth year	0.001	0.000	0.001	0.135	−0.001	−0.002	0.000	0.013
	Quadratic change * birth year	0.000	0.000	0.000	0.286				
	Intercept	−0.455	−0.500	−0.411	< 0.001	−0.269	−0.304	−0.234	< 0.001
	*Slope variance*	*0.001*	*0.001*	*0.001*		*0.061*	*0.058*	*0.064*	
	*Intercept variance*	*0.355*	*0.336*	*0.374*		*0.683*	*0.659*	*0.709*	
	*Slope-intercept covariance*	−*0.009*	−*0.011*	−*0.008*		−*0.119*	−*0.126*	−*0.112*	
Locomotor	Linear change	−0.044	−0.060	−0.029	< 0.001	−0.020	−0.032	−0.007	0.003
	Quadratic change	−0.001	−0.002	0.000	0.175				
	Birth year	0.048	0.045	0.051	< 0.001	0.034	0.032	0.036	< 0.001
	Linear change * birth year	0.001	0.000	0.002	0.011	0.003	0.003	0.004	< 0.001
	Quadratic change * birth year	0.000	0.000	0.000	0.891				
	Intercept	−0.719	−0.770	−0.668	< 0.001	−0.597	−0.632	−0.562	< 0.001
	*Slope variance*	*0.001*	*0.001*	*0.001*		*0.045*	*0.043*	*0.048*	
	*Intercept variance*	*0.420*	*0.396*	*0.446*		*0.529*	*0.506*	*0.552*	
	*Slope-intercept covariance*	−*0.011*	−*0.013*	−*0.009*		−*0.093*	−*0.100*	−*0.087*	
Vitality	Linear change	−0.020	−0.032	−0.008	0.001	−0.065	−0.079	−0.052	< 0.001
	Quadratic change	0.000	−0.001	0.000	0.196				
	Birth year	0.043	0.040	0.045	< 0.001	0.037	0.034	0.039	< 0.001
	Linear change * birth year	−0.001	−0.002	0.000	0.001	0.003	0.002	0.003	< 0.001
	Quadratic change * birth year	0.000	0.000	0.000	0.002				
	Intercept	−0.576	−0.619	−0.533	< 0.001	−0.498	−0.533	−0.463	< 0.001
	*Slope variance*	*0.000*	*0.000*	*0.000*					
	*Intercept variance*	*0.280*	*0.265*	*0.296*		*0.409*	*0.398*	*0.421*	
	*Slope-intercept covariance*	−*0.006*	−*0.007*	−*0.005*					
Cognition	Linear change	−0.027	−0.044	−0.011	0.001	−0.089	−0.102	−0.077	< 0.001
	Quadratic change	−0.003	−0.004	−0.002	< 0.001				
	Birth year	0.042	0.038	0.045	< 0.001	0.032	0.030	0.035	< 0.001
	Linear change * birth year	0.002	0.001	0.003	< 0.001	0.002	0.001	0.003	< 0.001
	Quadratic change * birth year	0.000	0.000	0.000	0.316				
	Intercept	−0.673	−0.730	−0.616	< 0.001	−0.370	−0.404	−0.335	< 0.001
	*Slope variance*	*0.002*	*0.001*	*0.002*		*0.062*	*0.059*	*0.065*	
	*Intercept variance*	*0.388*	*0.366*	*0.413*		*0.683*	*0.654*	*0.714*	
	*Slope-intercept covariance*	−*0.008*	−*0.010*	−*0.006*		−*0.116*	−*0.125*	−*0.108*	
Sensory	Linear change	−0.013	−0.031	0.004	0.139	0.015	0.002	0.029	0.025
	Quadratic change	−0.002	−0.003	−0.001	0.003				
	Birth year	0.024	0.020	0.027	< 0.001	0.022	0.019	0.025	< 0.001
	Linear change * birth year	0.000	−0.001	0.001	0.412	−0.001	−0.002	−0.001	0.001
	Quadratic change * birth year	0.000	0.000	0.000	0.119				
	Intercept	−0.382	−0.443	−0.320	< 0.001	−0.298	−0.336	−0.260	< 0.001
	*Slope variance*	*0.002*	*0.002*	*0.002*		*0.062*	*0.059*	*0.066*	
	*Intercept variance*	*0.489*	*0.462*	*0.518*		*0.719*	*0.691*	*0.748*	
	*Slope-intercept covariance*	−*0.018*	−*0.021*	−*0.016*		−*0.133*	−*0.142*	−*0.125*	

*Note*. Coef. and 95% Conf. Interval denote the coefficient and its 95% confidence intervals from multilevel growth curve models. For ELSA, longitudinal individual weights were used for multilevel growth curve models to ensure representativeness to participants aged 50 + and living in England in 2002 and still alive and residing in private households by wave 9. For CHARLS, survey weights were used for multilevel growth curve models to restore representativeness to participants aged 50 + and living in China. Random effects are included in italics.

**Table 3:** Scores by birth cohort and age in ELSA (1940 and 1950 cohorts)

	INTRINSIC CAPACITY						LOCOMOTOR CAPACITY					
	1950sCohort		1940s Cohort				1950s Cohort		1940s Cohort			
Age	Mean	Lower	Higher	Mean	Lower	Higher	Mean	Lower	Higher	Mean	Lower	Higher
**52**	0.665	0.619	0.711				0.71	0.662	0.758			
**54**	0.642	0.609	0.675				0.678	0.644	0.713			
**56**	0.612	0.586	0.638				0.641	0.614	0.667			
**58**	0.575	0.551	0.599				0.597	0.573	0.622			
**60**	0.531	0.506	0.555				0.548	0.522	0.573			
**62**	0.479	0.454	0.504	0.208	0.183	0.233	0.492	0.466	0.517	0.233	0.208	0.259
**64**	0.42	0.394	0.446	0.164	0.145	0.183	0.43	0.404	0.455	0.182	0.163	0.201
**66**	0.354	0.327	0.381	0.113	0.098	0.129	0.361	0.334	0.389	0.125	0.109	0.141
**68**	0.280	0.248	0.313	0.055	0.04	0.07	0.287	0.254	0.32	0.062	0.047	0.077
**70**				−0.011	−0.026	0.005				−0.007	−0.023	0.009
**72**				−0.084	−0.099	−0.068				−0.082	−0.098	−0.066
**74**				−0.164	−0.18	−0.148				−0.163	−0.179	−0.147
**76**				−0.252	−0.269	−0.235				−0.249	−0.267	−0.231
**78**				−0.347	−0.368	−0.326				−0.342	−0.364	−0.319
	VITALITY						COGNITIVE CAPACITY					
Age	1950s Cohort		1940s Cohort				1950s Cohort		1940s Cohort			
**52**	0.709	0.663	0.754				0.573	0.516	0.629			
**54**	0.609	0.576	0.641				0.611	0.573	0.65			
**56**	0.518	0.493	0.542				0.635	0.606	0.664			
**58**	0.437	0.415	0.458				0.644	0.618	0.671			
**60**	0.365	0.344	0.386				0.638	0.61	0.666			
**62**	0.303	0.282	0.324	0.280	0.255	0.306	0.618	0.588	0.647	0.157	0.126	0.189
**64**	0.251	0.23	0.272	0.2	0.181	0.218	0.582	0.551	0.613	0.162	0.14	0.183
**66**	0.208	0.186	0.23	0.124	0.109	0.138	0.532	0.498	0.566	0.149	0.132	0.166
**68**	0.174	0.148	0.201	0.053	0.04	0.066	0.467	0.425	0.508	0.12	0.103	0.136
**70**				−0.013	−0.025	0				0.074	0.056	0.091
**72**				−0.073	−0.086	−0.061				0.011	−0.007	0.029
**74**				−0.129	−0.141	−0.117				−0.069	−0.088	−0.05
**76**				−0.179	−0.193	−0.166				−0.165	−0.187	−0.144
**78**				−0.225	−0.242	−0.208				−0.278	−0.306	−0.251
	PSYCHOLOGICAL CAPACITY						SENSORY CAPACITY					
Age	1950s Cohort		1940s Cohort				1950s Cohort		1940s Cohort			
**52**	0.153	0.103	0.204				0.332	0.268	0.397			
**54**	0.172	0.137	0.207				0.327	0.284	0.371			
**56**	0.183	0.156	0.209				0.319	0.287	0.35			
**58**	0.186	0.162	0.21				0.307	0.279	0.335			
**60**	0.181	0.157	0.206				0.292	0.264	0.32			
**62**	0.169	0.144	0.195	−0.05	−0.079	−0.02	0.274	0.245	0.303	0.094	0.058	0.13
**64**	0.15	0.125	0.175	−0.042	−0.063	−0.022	0.252	0.223	0.281	0.08	0.055	0.104
**66**	0.123	0.096	0.149	−0.044	−0.061	−0.028	0.227	0.195	0.259	0.059	0.04	0.077
**68**	0.088	0.056	0.119	−0.056	−0.071	−0.041	0.199	0.157	0.24	0.031	0.014	0.048
**70**				−0.076	−0.092	−0.061				−0.004	−0.021	0.014
**72**				−0.106	−0.122	−0.091				−0.045	−0.063	−0.027
**74**				−0.145	−0.161	−0.13				−0.092	−0.111	−0.074
**76**				−0.194	−0.21	−0.177				−0.147	−0.167	−0.126
**78**				−0.251	−0.272	−0.231				−0.208	−0.235	−0.18

## Data Availability

Data described in this paper are available at the website of the English Longitudinal Study of Ageing (ELSA, https://www.elsa-project.ac.uk/) and the China Health and Retirement Longitudinal Study (CHARLS, http://charls.pku.edu.cn/en/). Ethical approval for all the ELSA waves was granted by NHS Research Ethics Committees under the National Research and Ethics Service (NRES). The Biomedical Ethics Review Committee of Peking University approved the CHARLS study (IRB00001052–11015). Furthermore, the current study received approval from the UNSW Ethics Committee (HC210472). The code scripts used in this study are available from the corresponding authors upon reasonable request.
